# Recent advances in understanding & managing male infertility

**DOI:** 10.12688/f1000research.9375.1

**Published:** 2016-11-24

**Authors:** Jared M. Bieniek, Kirk C. Lo

**Affiliations:** 1 Department of Urology, Hartford Hospital, 85 Seymour St., #416, Hartford, CT, 06106, USA; 2 Division of Urology, Mount Sinai Hospital, University of Toronto, 60 Murray St., 6th floor, Box 19, Toronto, ON, M5T3L9, Canada

**Keywords:** infertility, male, idiopathic infertility

## Abstract

Male infertility remains a struggle to definitively diagnose and treat with many men labelled as “idiopathic infertility” and eventually requiring assisted reproductive techniques.  Along those lines, research groups are continuing to explore current social and environmental factors, including the obesity epidemic, and their effects on male fertility potential.  Novel biomarkers of natural fertility status and azoospermia etiology have additionally seen recent attention with ACRV1 and TEX101/ECM1 assays either currently or soon to be commercially available.  Despite these advancements, however, medical treatment options have seen little progress.  Though surgical therapies have similarly seen little transformation, groups are exploring the use of testicular sperm for couples with elevated sperm DNA fragmentation and either planned or previously failed IVF/ICSI.  Concerted collaborative efforts will be needed as we move forward to better understand the challenges men face when struggling to conceive.

## Introduction

Ongoing research efforts strive to better define the largely “idiopathic” world of male infertility. Up to 30% of infertile men are labeled with idiopathic sperm abnormalities ^1^, and before personalized treatment recommendations can be made for these men, we need to identify the causes of infertility. Along those lines, groups are taking an “-omics” approach, exploring genomics, proteomics, transcriptomics, and metabolomics in an effort to discover better male fertility biomarkers. Others have redirected their efforts to look at current lifestyle factors and their impact on today’s infertility rates. Several studies have demonstrated an ominous decline in overall sperm quantity and quality in the last several decades, with some blaming the obesity epidemic as the cause ^2, 3^. Large multi-institutional studies are being designed to analyze body habitus and other current social and environmental factors and their relationship with male fecundity.

Medical treatment options for men trying to conceive represent another area in great need of further exploration. While the market has seen the addition of three new testosterone formulations over the past two years, no FDA-approved medications exist for the treatment of male infertility other than injectable gonadotropin formulations. Certainly the broad male hypogonadism, or “low T”, marketing strategy has attracted the public’s attention and, with it, pharmaceutical interest. Moving forward, it will be interesting to see if the public appeal of testosterone replacement therapy (TRT) will lead to younger men seeking TRT with resultant infertility.

Similar to the popularity of TRT, the men’s health movement has gained great traction over the past several years. We advocate for male fertility evaluation as an essential element of the general health assessment of men of reproductive age, as recommended in the American Urological Association’s (AUA) Men’s Health Checklist ^4^. The importance of recognizing male infertility as a medical condition has been made evident in recent studies noting the association between male infertility and future risks of testicular cancer, diabetes, and heart disease ^5^. In this review, we describe specific advancements in research on lifestyle factors and male fertility, novel infertility biomarkers, and management of elevated sperm DNA fragmentation in assisted reproductive treatment. Recent advancements in the field such as these have begun to further clarify etiologies and treatments for male infertility, with a great need for future research and advocacy.

## Recent advances

### Lifestyle factors and male fertility

Along with the recognition of our poor understanding of male infertility in certain cases, many have recognized that social and environmental factors studied in decades past may not apply to today’s reproductive-aged population. Much of the past research was also performed on retrospective data sets with their own inherent biases and limitations. The Longitudinal Investigation of Fertility and the Environment (LIFE) study was designed to be one of the first prospective studies to analyze fertility factors in couples of unknown fertility status ^6^. To do this, 501 couples were recruited from two geographic areas (Texas and Michigan) as they initiated their efforts to conceive. The couples underwent intensive screening before and during their attempts to become pregnant over a one-year period. In total, 94% (n=473) of the male cohort provided a semen sample for analysis and 80% provided a second sample.

For those men providing a semen sample in the LIFE study, their data were correlated to various lifestyle factors and reported in two publications. In a 2015 report, the study authors detailed the negative effects of heavy occupational exertion (sperm concentration and total count), hypertension (strict morphology), and increasing total number of medications (sperm count) ^7^. Their second publication focused largely on correlations between semen quality and measures of obesity, with 82% of the overall male cohort being overweight or obese (body mass index [BMI] ≥25) at baseline ^8^. Findings included a linear decline in ejaculate volume associated with increasing BMI and waist circumference (WC). WC was also noted to have a negative relationship with total sperm count (TSC); no significant correlations with sperm concentration, motility, morphology, DNA integrity, or vitality were found. Overall, an increasing frequency of men with abnormal ejaculate volume, sperm concentration, and TSC were seen with increasing body size, though, demonstrating correlations in a population of men without known infertility.

Determining the fertility consequences of a man’s weight and metabolic status is essential given new statistics showing ever-swelling waistlines of the American male population with 35% currently estimated to be obese ^9^. While obesity results in dependable hormonal alterations, including a decrease in the testosterone/estradiol (T/E) ratio, contradictory effects on seminal parameters have been found, as shown by two discrepant meta-analyses on the topic. In 2010, the first meta-analysis assessed data from five studies and over 4,800 men, concluding that no significant relationships existed between BMI and semen parameters ^10^. An updated review in 2013, however, reported increased prevalence of oligospermia or azoospermia in overweight and obese men from an analysis of over 13,000 men ^11^.

Many of the included studies in these meta-analyses, however, were small single-center retrospective series. In an effort to bolster the literature and include a larger obese cohort, three North American male infertility centers recently combined their prospectively collected data to analyze BMI and its relationship with semen and reproductive hormonal parameters ^12^. Of 4,440 men included, 45.1% were overweight and 23.3% were obese at the time of initial evaluation. BMI negatively impacted reproductive hormones, as expected, with the greatest absolute effect on T/E ratio. All semen parameters were found to have negative correlations with BMI with significance noted for ejaculate volume, sperm concentration, morphology, and total motile count on multivariate analyses. Relative risks of oligospermia and azoospermia among the obese group, as compared to normal BMI, were 1.34 (95% confidence interval: 1.18–1.53) and 1.44 (1.14–1.81), respectively.

If weight appears to have real effects on male fertility, the next logical question would be whether weight loss strategies result in semen quality improvement. In an initial study of exercise and diet-based weight loss regimens, promising changes in male reproductive parameters were seen ^13^. Certainly this topic warrants further exploration with additional appraisal of male fertility changes associated with the drastic weight loss of bariatric surgery.

### Novel biomarkers

Much of our clinical evaluation and research efforts of male fertility depend on a single test: the semen analysis. Despite its use as the “gold standard”, the semen analysis remains an imprecise measure of male fertility potential with significant intra-individual variability and large overlapping values between groups of fertile and infertile men. DNA fragmentation index (DFI) has also been studied extensively as a male fertility biomarker as described in a previous review ^14^. In addition to DNA, sperm also deliver a complex profile of proteins, RNA, and other molecules to the oocyte necessary for proper fertilization and development. An ever-increasing number of research groups are utilizing novel technologies to investigate each of these “-omics” areas in an effort to discover novel male fertility biomarkers. Transcriptomics, for example, has had promising results from several groups, demonstrating RNA profiles of “required elements” for natural fertility ^15, 16^.

While various bodily fluids can be sampled to investigate novel fertility biomarkers, seminal plasma contains concentrated levels of proteins derived from the male reproductive system and may prove the most fruitful. Batruch *et al*. were able to identify over 2,300 individual proteins from semen samples of fertile and infertile men using mass spectrometry ^17^. Groups are using these available data from various cohorts to better assess natural fertility and azoospermia etiologies ( Table 1). In one such study, prostaglandin D synthase (PGDS) levels were found to positively correlate with sperm concentration, motility, and morphology ^18^. While other groups have started to compare seminal plasma proteomic profiles between small cohorts of fertile and infertile men ^19, 20^, larger collaborative studies are needed before any results can be validated for possible clinical applications.

**Table 1.** Recently reported human male infertility biomarkers.

Protein	Gene	Diagnostic application	Year
Acrosomal vesicle protein 1	ACRV1	Azoospermia	2008 ^38^
Prostaglandin D synthase	PGDS	NOA versus OA	2008 ^39^
Prolactin-inducible protein	PIP	Azoospermia	2012 ^40^
Cathelicidin antimicrobial peptide	CAMP	NOA versus OA	2013 ^23^
Extracellular matrix protein 1	ECM1	NOA versus OA	2013 ^23^
L-lactate dehydrogenase C chain	LDHC	Azoospermia	2013 ^23^
Lectin galactoside-binding soluble 3 binding protein	LGALS3BP	Testicular sperm extraction outcome	2013 ^41^
Testis expressed 101	TEX101	NOA subtypes	2013 ^23^
Transketolase-like protein 1	TKTL1	Azoospermia	2013 ^42^

Abbreviations: NOA, non-obstructive azoospermia; OA, obstructive azoospermia.

Another area in need of additional clinical diagnostics is the discrimination between obstructive azoospermia (OA) and non-obstructive azoospermia (NOA) for proper patient counseling and management in select circumstances of undetermined azoospermia. Follicle-stimulating hormone (FSH) and testicular size are commonly used to predict between the two, though with limited sensitivity in some men ^21^. Testicular biopsy is necessary if one wishes to differentiate subtypes of NOA. Efforts from various research groups have identified a number of potential protein biomarkers including PGDS, acrosomal vesicle protein 1 (ACRV1), lectin galactoside-binding soluble 3 binding protein (LGALS3BP), extracellular matrix protein 1 (ECM1), and testis expressed 101 (TEX101) ( Table 1) ^22^. ACRV1, in particular, has already been incorporated into two commercially available tests for general fertility and post-vasectomy assessments. Drabovich *et al*. further reviewed candidate proteins from seminal protein profiles of subgroups of men to discriminate between OA and NOA as well as between pathologic subtypes of NOA, the knowledge of which can aid in predicting microscopic testicular sperm extraction success ^23^. ECM1, an epididymal protein, was able to assess for vasal patency and discriminate between NOA and OA with 73% specificity at 100% sensitivity. TEX101, a testicular protein, aided in differentiating hypospermatogenesis, maturation arrest, and Sertoli cell-only patterns of NOA. A combined assay using ECM1 and TEX101 is currently under development.

Men with NOA should also have initial genetic testing with a karyotype and Y chromosome microdeletion assay, together revealing abnormalities in approximately 20% of men ^24^. This figure likely underestimates the true prevalence of genetic aberrations in male infertility as groups discover new genetic biomarkers. The review by Kovac *et al*. nicely summarizes a number of potential gene biomarkers reported in the literature ^25^. Additionally, TEX11 mutations of the X chromosome were recently noted in an array comparative genomic hybridization study, affecting seven of 289 (2.4%) screened men with NOA ^26^. TEX11 codes for a testis-specific meiotic protein that regulates DNA double-strand break repair and has proven essential to normal spermatogenesis in mouse models ^27^. Continued investigatory efforts involving large groups of infertile men will be needed as we work to define genomic and other etiologies of infertility in this heterogeneous population.

### Management of elevated DNA fragmentation

One of the few male infertility treatment areas that have seen advances in the past several years has been the management of elevated DNA fragmentation index (DFI) in *in vitro* fertilization (IVF)/intracytoplasmic sperm injection (ICSI). Based on the concept that much of the DNA damage in ejaculated sperm occurs at the epididymal level, Greco *et al*. began exploring the use of surgically retrieved testicular sperm in couples with elevated DFI ^28^. In 18 couples who had previously failed ICSI with ejaculated sperm, repeat ICSI was performed using testicular sperm. Sperm DFI rates proved to be much lower in the testicular samples and eight of 18 (44.4%) couples were able to achieve a pregnancy with testicular sperm.

The use of testicular sperm to optimize outcomes in couples with failed fertility attempts has gained popularity with several recent publications and research presentations ^29^. A recent review of 147 couples undergoing IVF with elevated sperm DFI levels (>30% on sperm chromatin dispersion assay despite oral antioxidant therapy) revealed significant reductions in DFI using testicular sperm over ejaculated specimens (8.3% and 40.7%, respectively) ^30^. Significant improvements in clinical pregnancy (51.9% versus 40.2%), miscarriage (10.0% versus 34.3%), and live birth (46.7% versus 26.4%) rates were also seen for the testicular-ICSI versus ejaculated-ICSI groups, respectively. The 2016 AUA meeting saw two additional presentations on this timely topic. Presenting on the Qatar experience, Al-Malki described the use of testicular sperm for 36 couples with high DFI (>30% on Halosperm assay) and prior IVF-ICSI cycles ^31^. While no differences were noted in fertilization rate or embryo grading, clinical pregnancy rates were significantly higher (38.9% versus 13.8%). A similar report by Patel *et al*. detailed outcomes for 44 couples with elevated DFI (>24% on sperm chromatin structure assay) undergoing ICSI with sperm obtained via testicular sperm aspiration ^32^. Only 28 of the couples had failed prior ICSI or had a miscarriage. Overall pregnancy rate was reported at 38.6% and even slightly higher in the cohort with prior failed ICSI or miscarriage (42.9%). In contrast to prior studies, Patel *et al*. reported higher fertilization and embryo quality rates with testicular versus ejaculated sperm. Many groups are now investigating the use of testicular sperm for couples with elevated sperm DFI with promising pregnancy, live birth, and miscarriage rates ^33– 35^. It is important to stress that testicular sperm may not improve IVF/ICSI outcomes in all couples, as shown in a recent meta-analysis of men with cryptozoospermia (sperm DFI not reported) ^36^. Clearly larger studies with longitudinal follow-up are needed to better characterize the role of testicular sperm retrieval for assisted reproductive techniques.

## Clinical implications and future directions

Larger studies with longer follow-up are necessary to define our evaluation and treatment of men struggling to conceive. Female fertility outcomes are tracked nationally with required reporting to the Society for Assisted Reproductive Technology database. The Andrology Research Consortium (ARC) was founded in 2013 in collaboration with the Society for the Studies of Male Reproduction along these lines to advance the field of male fertility research through expansive collaborative data. Fourteen North American centers are currently participating in the collection of initial intake data from men presenting for infertility investigation.

The initial data collected as part of ARC was presented at the AUA 2016 annual meeting, including information from 2,108 men, 84% of whom presented with primary infertility ^37^. Approximately 3% of men admitted to exogenous testosterone use with rates as high as 10% at some centers. Of couples who had previously undergone intrauterine insemination (IUI) or IVF cycles, disturbingly few reported a prior male fertility evaluation (9.8% and 28%, respectively). Future efforts will aim to better define the role of medical and lifestyle factors, including obesity, on male fertility status among an even larger multi-institutional cohort. With additional data collection, we may be able to better understand etiologies of male factor infertility and appropriate treatment choices. Currently, our ever-expanding research on infertility demonstrates the complexity of the human body, and, hopefully, with concerted collaborative efforts we will begin to better understand the challenges our patients face in trying to become parents.

## Abbreviations

ACRV1, acrosomal vesicle protein 1; ARC, Andrology Research Consortium; AUA, American Urological Association; BMI, body mass index; DFI, DNA fragmentation index; ECM1, extracellular matrix protein 1; ICSI, intracytoplasmic sperm injection; IVF, *in vitro* fertilization; LIFE, Longitudinal Investigation of Fertility and the Environment; NOA, non-obstructive azoospermia; OA, obstructive azoospermia; PGDS, prostaglandin D synthase; T/E ratio, testosterone/estradiol ratio; TEX101, testis expressed 101; TRT, testosterone replacement therapy; TSC, total sperm count; WC, waist circumference.
